# Diaphragm neurostimulation reduces mechanical power and mitigates brain injury associated with MV and ARDS

**DOI:** 10.1186/s40001-022-00932-4

**Published:** 2022-12-19

**Authors:** Thiago G. Bassi, Elizabeth C. Rohrs, Karl C. Fernandez, Marlena Ornowska, Michelle Nicholas, Matt Gani, Doug Evans, Steven C. Reynolds

**Affiliations:** 1 Lungpacer Medical Inc, Vancouver, BC Canada; 2 Simon Fraser University, Burnaby, BC Canada; 3 Fraser Health Authority, Royal Columbian Hospital, New Westminster, BC Canada

**Keywords:** Diaphragm neurostimulation, Diaphragm pacing, Mechanical power, Ventilator-induced lung injury, Ventilation-associated brain injury

We read the recently published review by Ziaka and Exadaktylos with great enthusiasm. The authors summarized the current knowledge regarding brain injury associated with acute respiratory distress syndrome (ARDS) extremely well [1]. They described the accepted mechanisms by which ARDS might result in brain injury and possibly acute and chronic cognitive impairment [1]. Among the mechanisms described were systemic inflammation, hypoxemia, and adverse effects of mechanical ventilation (MV) [1]. According to Ziaka and Exadaktylos’s review, the interaction between the patient and the ventilator might lead to brain injury due to individual susceptibility to the development of acute lung injury during MV [1]. The authors postulated that this is a consequence of a disruption in the neural control of respiration and immunological response due to lung injury associated with ARDS [1]. The review stated that one possible cause of brain injury due to ARDS is a disruption in the conversion of the mechanical stimuli produced by the pulmonary stretch receptors during MV, which in turn results in an aberrant biological signal triggering brain injury [1]. The review also pointed out that systemic inflammation, blood–brain barrier leakage, and hypoxemia might contribute to brain injury secondary to ARDS [1]. Regardless of the mechanism that triggered brain injury the clinical consequence is cognitive impairment, acute and chronic, and delirium [1]. Although many lung-protective ventilatory strategies, such as low tidal volume and PEEP optimization, have been put in place to reduce systemic inflammation and injury, ventilator-induced injury still contributes to morbidity and mortality in this population [1]. Ziaka and Exadaktylos propose that as MV and ARDS are associated with brain injury, a neuroprotective ventilatory strategy for patients with otherwise normal brains is an area that needs further investigation [1].

Our group recently published results reporting that lung-protective MV for 50 h, in a non-injured-lung pig model, was associated with brain injury when compared to a never-ventilated group [2]. We demonstrated that animals that received protective ventilation (volume control, tidal volume 8 ml/kg, PEEP 5 cmH_2_O) developed brain injury, and that combining a novel ventilatory strategy utilizing transvenous diaphragm neurostimulation (TTDN) with the same protective ventilation provided neuroprotection [2]. This novel intervention utilizes a central line catheter, embedded with electrodes, which bilaterally stimulates the phrenic nerves to contract the diaphragm in synchrony with the inspiratory phase of MV [2–4]. In our study, TTDN settings targeted a reduction in the ventilator pressure–time product of 15–20% [2–4]. To isolate MV as an independent variable, we studied pigs with non-injured lungs. Three experimental groups: MV only, MV + TTDN every other breath (MV + TTDN50%), and MV + TTDN every breath (MV + TTDN100%), were mechanically ventilated, as defined above, for 50 h. Never-ventilated subjects were used as a control group. All three mechanically ventilated groups received intravenous sedation per a standardized protocol employing propofol, midazolam, ketamine, and fentanyl [2–4]. There were no significant differences in sedative drug doses between groups [2–4].

The TTDN-every-breath group showed levels of neuroinflammation as measured by hippocampal microglia percentage that were statistically indistinguishable from the never-ventilated group [4]. The group receiving MV-only showed considerably higher levels of neuroinflammation compared to both groups receiving MV + TTDN [4]. Ziaka and Exadaktylos conclude that systemic inflammatory cascades contribute significantly to neuroinflammation and that protective MV can mitigate this response [1]. In our study, systemic inflammation was measured at study end. There was no statistically significant difference between median (IQR) serum concentrations of IL-1β [154 pg/ml (69–458) MV; 100 pg/ml (18–136) MV + TTDN50%; 182 (115–264) MV + TTDN100%; *p* = 0.138] or IL-6 [45 pg/ml (25–216) MV; 18 pg/ml (0–216) MV + TTDN50%; 127 (78–180) MV + TTDN100%; *p* = 0.010] in the mechanically ventilated groups. Systemic inflammation is not likely to be a significant cause of the difference in neuroprotection between the groups in our study [3, 4]. Our study did demonstrate a moderate correlation between mechanical power (a measure of the energy delivered over time by the mechanical ventilator) and hippocampal microglia percentage. Total mechanical power was calculated as the area under the curve using the formula MP = 0.098*RR*VT*(PIP-½(driving pressure)).^5^ Total study exposures to mechanical power measured were, respectively [total area under the curve (std. error)]: 560 J/min*hours (25) MV; 488 J/min*hours (22) MV + TTDN50%, 376 J/min*hours (10) MV + TTDN100%. Microglia percentages found were [median (interquartile range)]: 34% (32–40) MV, 17% (12–23) MV + TTDN50%, and 10% (8–11) MV + TTDN100%. Spearman correlation test showed a positive, linear, and moderate correlation between hippocampal microglia percentages and total study exposure to mechanical power (*r* = 0.49, *p* = 0.03). These results isolate MV itself as an important variable in the development of neuroinflammation, and implicate mechanical power as a key element in the occurrence of hippocampal inflammation associated with MV.

Our group more recently investigated whether TTDN would also promote the mitigation of ventilation-associated brain injury in a 12-h moderate-ARDS preclinical model. ARDS was induced by injecting oleic acid into the pulmonary artery [6]. Lung-protective MV was delivered to three experimental groups: MV only, MV + TTDN every other breath (50%), and MV + TTDN every breath (100%), with ventilator settings and TTDN, as defined above, delivered for 12 h [6]. Mechanical power at study-end and microglia percentages were, respectively [median (IQR)]: 15 J/min (13–17) and 18% (17–32) LI-MV; 11 J/min (10–14) and 13% (12–14), LI-MV + TTDN50%; 9 J/min (6–12) and 9% (8–10) LI-MV + TTDN100% [5, 6]. Spearman correlation test again showed a positive, linear, and moderate correlation between hippocampal microglia percentages and mechanical power, *r* = 0.55, *p* = 0.02. These results combined with the results from our 50-h non-injured lung study support the hypothesis that there are alternative pathways of neuroprotection that are activated when protective MV is combined with TTDN in a moderate ARDS model.

This letter complements Ziaka and Exadaktylos’s review by showing preclinical data from a novel ventilatory neuroprotective intervention that might help to mitigate ventilation-associated brain injury and ARDS-associated brain injury. This work elucidates that ventilator energy transferred to lung tissue with or without ARDS during MV plays an important role in this process. More work is needed to identify the specific pathways responsible for these results, as the question of whether the findings reported here result from direct neural signals or circulating factors remains to be determined.

## Data Availability

All the data are available upon reasonable request.
